# Rare-Earth Oxides as Alternative High-Energy Photon Protective Fillers in HDPE Composites: Theoretical Aspects

**DOI:** 10.3390/polym13121930

**Published:** 2021-06-10

**Authors:** Kiadtisak Saenboonruang, Worawat Poltabtim, Arkarapol Thumwong, Theerasarn Pianpanit, Chanis Rattanapongs

**Affiliations:** 1 Department of Applied Radiation and Isotopes, Faculty of Science, Kasetsart University, Bangkok 10900, Thailand; wp.worawat@gmail.com (W.P.); arkarapol.th@ku.th (A.T.); fscitap@ku.ac.th (T.P.); fscicnp@ku.ac.th (C.R.); 2 Specialized Center of Rubber and Polymer Materials in Agriculture and Industry (RPM), Faculty of Science, Kasetsart University, Bangkok 10900, Thailand

**Keywords:** HDPE, Sm_2_O_3_, Eu_2_O_3_, Gd_2_O_3_, photon, gamma, X-ray, shielding, XCOM

## Abstract

This work theoretically determined the high-energy photon shielding properties of high-density polyethylene (HDPE) composites containing rare-earth oxides, namely samarium oxide (Sm_2_O_3_), europium oxide (Eu_2_O_3_), and gadolinium oxide (Gd_2_O_3_), for potential use as lead-free X-ray-shielding and gamma-shielding materials using the XCOM software package. The considered properties were the mass attenuation coefficient (µ_m_), linear attenuation coefficient (µ), half value layer (HVL), and lead equivalence (Pb_eq_) that were investigated at varying photon energies (0.001–5 MeV) and filler contents (0–60 wt.%). The results were in good agreement (less than 2% differences) with other available programs (Phy-X/PSD) and Monte Carlo particle transport simulation code, namely PHITS, which showed that the overall high-energy photon shielding abilities of the composites considerably increased with increasing rare-earth oxide contents but reduced with increasing photon energies. In particular, the Gd_2_O_3_/HDPE composites had the highest µ_m_ values at photon energies of 0.1, 0.5, and 5 MeV, due to having the highest atomic number (Z). Furthermore, the Pb_eq_ determination of the composites within the X-ray energy ranges indicated that the 10 mm thick samples with filler contents of 40 wt.% and 50 wt.% had Pb_eq_ values greater than the minimum requirements for shielding materials used in general diagnostic X-ray rooms and computerized tomography rooms, which required Pb_eq_ values of at least 1.0 and 1.5 mmPb, respectively. In addition, the comparisons of µ_m_, µ, and HVL among the rare-earth oxide/HDPE composites investigated in this work and other lead-free X-ray shielding composites revealed that the materials developed in this work exhibited comparable X-ray shielding properties in comparison with that of the latter, implying great potential to be used as effective X-ray shielding materials in actual applications.

## 1. Introduction

High-energy photon technologies, especially those related to X-rays and gamma rays, have been extensively used in several applications such as X-ray and gamma imaging for medical diagnostic and material characterization [1,2,3,4], gamma-induced mutation breeding in plants [5,6], quality control and quality assurance for industrial products [7], and gemstone irradiation for color enhancement [8,9]. Despite the great benefits of such applications, excessive exposure to high-energy photons could severely harm users and others, with the effects varying from mild symptoms (rash, skin burn, nausea, and headache) to fatal diseases (cancers and genetic mutations) that could cause permanent disabilities or possible death [10,11]. To reduce and/or prevent such risks, a radiation safety principle called As Low As Reasonably Achievable (ALARA), must be strictly followed in all nuclear-related facilities that consist of proper time management, operational distance, and use of shielding equipment [12,13].

Particularly for shielding, the development of novel materials that offer not only enhanced shielding capabilities but also additionally preferred properties, such as exceptional strength and being environmentally friendly, has become a necessity in the fast-growing radiation technology to ensure the highest safety for all related personnel and users. Generally, materials containing heavy metals, especially lead (Pb), are commonly used to attenuate high-energy photons due to Pb being economically accessible and having a relatively high atomic number (Z = 82) and density (ρ = 11.34 g/cm^3^), which greatly enhances the interaction probability of the materials with incident photons that result in superior high-energy photon shielding properties [14,15]. Examples of Pb-containing materials used for high-energy photon shielding are Pb-borate glasses doped with aluminum oxide [16], Pb-fly ash concrete [17], and nano-PbO/EPDM composites [18]. However, it has been proven that Pb and Pb-containing materials have serious drawbacks due to their toxicity, with prolonged exposure to Pb being potentially harmful to almost every important organ and part of humans, animals, and plants [19,20]. To reduce such health concerns, new and safe Pb-free shielding materials have been constantly pursued, such that the developed materials offer better photon attenuation and are safer for production and use. Examples of reported Pb-free shielding materials are self-healable Bi_2_O_3_/PVA hydrogels [21], flexible EPDM and NR composites containing Bi_2_O_3_, WO_3_, and Fe_3_O_4_ particles [22,23], and transparent Bi_2_O_3_/B_2_O_3_/BaO glasses [24], for which the efficiencies of the mentioned materials in photon attenuation varied depending on the filler type, content, and size [25].

In addition to the Bi, W, and Fe compounds commonly used as fillers for the production of Pb-free shielding materials, rare-earth oxides have gained considered attention from researchers and product developers, especially samarium oxide (Sm_2_O_3_), europium oxide (Eu_2_O_3_), and gadolinium oxide (Gd_2_O_3_), for use as alternative high-energy photon protective fillers, due to their relatively high densities (7.40–8.35 g/cm^3^) and atomic numbers (Z_Sm_ = 62, Z_Eu_ = 63, and Z_Gd_ = 64). Examples of rare-earth-oxide-containing materials, previously developed for use as high-energy photon shielding materials, are TeO_2_-ZnF_2_-As_2_O_3_-Sm_2_O_3_ glasses [26], waste soda-lime glass doped with La_2_O_3_ and Gd_2_O_3_ [27], Eu_2_O_3_-reinforced zinc-borate glasses [28], erbium (III)- and terbium (III)-containing silicate-based bioactive glass [29], and rare-earth/glassy alloys [30], which all had highly promising photon shielding ability. Another important advantage of rare-earth oxides (Sm_2_O_3_, Eu_2_O_3_, and Gd_2_O_3_) over the common Pb, Bi_2_O_3_, WO_3_, and Fe_2_O_3_ fillers is that the former are able to attenuate not only high-energy photons but also thermal neutrons with exceptional efficiency (even higher than common borated materials) [31]. These dual shielding properties of the materials containing Sm_2_O_3_, Eu_2_O_3_, or Gd_2_O_3_ are crucially useful for workers in proximity to nuclear facilities, such as nuclear reactors and ion accelerators that have a photon–neutron-mixed environment [32]. The superior neutron attenuation abilities of Sm_2_O_3_, Eu_2_O_3_, and Gd_2_O_3_ are due to their relatively high neutron absorption cross sections (σ_abs_) of the Sm, Eu, and Gd in the compounds, which are 5922, 4530, and 49,700 barns, respectively, compared to Bi, W, and Fe, which have much lower σ_abs_ values of 0.0338, 18.3, and 2.56 barns, respectively. The abilities of Sm_2_O_3_, Eu_2_O_3_, and Gd_2_O_3_ to simultaneously attenuate both high-energy photons and thermal neutrons, as well as being self-gamma attenuators, have led to considerably simpler material designs and fewer chemicals and processes needed to produce the individual shielding materials required for the attenuation of photons and neutrons. Examples of rare-earth-oxide-containing materials to attenuate neutron shielding are Sm_2_O_3_/PVA and Gd_2_O_3_/PVA composites [33], Sm_2_O_3_/UHMWPE composites [34], Sm_2_O_3_/HDPE and Gd_2_O_3_/HDPE composites [35], and ZnO-B_2_O_3_-TeO_2_-Eu_2_O_3_ glasses [36].

In addition to attenuation effects from fillers, the main matrices used for the production of the shielding materials also play an important role in defining mechanical properties of the materials. For example, in applications requiring high strength, such as structural parts (walls, partitions, and equipment enclosures) and transporting casks for nuclear sources, an HDPE having a chemical formula of (C_2_H_4_)_n_ is one of the preferred choices due to its superior tensile strength compared to other thermoplastics, its excellent electrical insulation, its low water absorption, and its good processibility [37,38]. As a result, HDPE composites filled with different radiation protective fillers have been continuously developed and used as shielding materials to achieve both mechanical strength and enhanced shielding properties. Examples of HDPE composites used in radiation protection are Bi_2_O_3_/HDPE [39] and ZnO/HDPE [40] for gamma shielding and CdO/HDPE [41] and h-BN/Gd_2_O_3_/HDPE [42] for neutron shielding.

As previously mentioned, the current work theoretically determined the high-energy photon shielding properties of Sm_2_O_3_/HDPE, Eu_2_O_3_/HDPE, and Gd_2_O_3_/HDPE composites using XCOM, based on the mass attenuation coefficient (µ_m_), linear attenuation coefficient (µ), half value layer (HVL), and lead equivalence (Pb_eq_) [43,44]. To fully understand the effects of filler types and contents, and incident photon energies on shielding properties, the contents of Sm_2_O_3_, Eu_2_O_3_, and Gd_2_O_3_ in HDPE composites were varied (0–60 wt.%) for a range of photon energies (0.001–5 MeV). To verify the correctness and reliability of the investigation, the µ values obtained from XCOM were compared with results from available online software (Phy-X/PSD) and Monte Carlo code (Particle and Heavy Ion Transport Code System; PHITS), and differences between their results were investigated. Furthermore, to assess the useability of the developed composites in actual applications, the Pb_eq_ for all samples were determined at photon energies of 0.06, 0.08, and 0.1 MeV (X-ray ranges) and the results were compared with the minimum requirements for use in general diagnostic X-ray rooms and CT rooms, which are 1 mmPb and 1.5 mmPb, respectively. Lastly, to benchmark the shielding ability of the materials from this work with other common Pb-free composites, such as HDPE composites containing Bi_2_O_3_, WO_3_, and Fe_2_O_3_, and glassy alloys containing rare-earth elements, values of µ_m_, µ, and HVL at filler contents of 20, 40, and 60 wt.% and photon energies of 0.1, 0.5, 1, and 5 MeV were compared and discussed. The outcomes of this work should not only reveal theoretically the effectiveness of rare-earth oxides to attenuate high-energy photons but also increase the availability of the currently limited information on the use of rare-earth oxides for radiation protection.

## 2. Determination of High-Energy Photon Shielding Properties

### 2.1. Determination of Mass Attenuation Coefficient (µ_m_)

The XCOM software provided by the National Institute of Standards and Technology (NIST) (Gaithersburg, MD, USA), was used to determine the values of µ_m_ in the HDPE composites filled with either rare-earth oxides (Sm_2_O_3_, Eu_2_O_3_, or Gd_2_O_3_) or common Pb-free fillers (Bi_2_O_3_, WO_3_, and Fe_2_O_3_) at filler contents of 0–60 wt.% and photon energies of 0.001–5 MeV. In addition, the µ_m_ values of a pure Pb sheet were also determined at photon energies of 0.06, 0.08, and 0.1 MeV to provide comparative X-ray shielding properties of rare-earth oxides/HDPE composites with respect to a standard Pb sheet. The photon cross-section database used in this work was the NIST standard reference database 8 (XGAM), released in November 2010. The values of µ_m_ reported in this work were calculated from the total attenuation with the inclusion of coherent scattering [45]. Furthermore, to be able to determine µ_m_ for any filler content, simple mathematical relationships were developed between µ_m_ and the filler content at the photon energies of 0.1, 0.5, 1, and 5 MeV in the form shown in Equation (1):

(1) $$\mu_{m}=\mathrm{Ax}+B$$

where µ_m_ is the mass attenuation coefficient, x is the filler content, and A (B) is the mathematical coefficient, determined using a trendline function available in the Microsoft Excel software package.

### 2.2. Verification of XCOM Results by Phy-X/PSD and PHITS

To verify the correctness and the reliability of the results obtained from XCOM for further investigation, other programs, namely Phy-X/PSD [46] and PHITS [47], were used to calculate the µ_m_ values of HDPE composites containing Sm_2_O_3_, Eu_2_O_3_, or Gd_2_O_3_ at filler contents of 20, 40, and 60 wt.% and photon energies of 0.1, 0.5, 1, and 5 MeV. In particular, for the determination of µ_m_ from PHITS, the Monte Carlo code (version 3.22) was set up such that the photon beam with a diameter of 1 mm was directed at the center of the sample, which had the surface area of 20 cm × 20 cm and the thickness of 1 cm, to minimize effects from build-up factor. Furthermore, the detector was set up in such a way that its size was the same as the photon beam and had 100% detection efficiency in order to capture all primary transmitted photons. More details of the setup for PHITS could be found in the previous reports of Toyen and Saenboonruang [25] and Poltabtim et al. [35] To verify the results obtained from this work, their results were compared and differences between the values were calculated using Equation (2):

(2) $$\mathrm{Difference}\;\left(\%\right)=\frac{\left|\mu_{m,\;\mathrm{XCOM}}-\mu_{m,\;\mathrm{ref}}\right|}{\mu_{m,\;\mathrm{XCOM}}}\times 100\%$$

where Difference (%) is the percentage difference between the µ_m_ values obtained from XCOM and Phy-X/PSD (PHITS), µ_m,XCOM_ is the mass attenuation coefficient obtained from XCOM, and µ_m,ref_ is the mass attenuation coefficient obtained from either Phy-X/PSD or PHITS.

### 2.3. Determination of Linear Attenuation Coefficient (µ) and Half Value Layer (HVL)

The values of µ and HVL of the HDPE composites, which represent the fraction of attenuated incident photons in a monoenergetic beam per unit thickness and the thickness of the materials required to attenuate 50% of incident photons, respectively, were determined from the obtained values of µ_m_ (XCOM) in Section 2.1 using Equations (3) and (4), respectively [43]:

(3) $$\mu =\mu_{m}\rho$$

(4) $$HVL=\frac{ln\left(2\right)}{\mu }$$

where ρ is the density of the composites theoretically estimated using Equation (5):

(5) $$\rho =\frac{100}{\frac{C_{\mathrm{HDPE}}}{\rho_{\mathrm{HDPE}}}+\frac{C_{F}}{\rho_{F}}}$$

where ρ_HDPE_ (ρ_F_) is the density of HDPE (radiation protective filler with individual densities shown in Table 1) and c_HDPE_ (c_F_) are the contents of the HDPE (radiation protective fillers). It should be noted that c_HDPE_ + c_F_ = 100 wt.%. Similar to µ_m_, simple mathematical relationships were developed between µ (HVL) and the filler content in the form shown in Equation (6):

(6) $$\mu \;\left(\mathrm{HVL}\right)={\mathrm{Ae}}^{\mathrm{Bx}}$$

where µ (HVL) is the linear attenuation coefficient (half value layer), x is the filler content, and A (B) is the mathematical coefficient, determined using a trendline function available in the Microsoft Excel software package.

### 2.4. Determination of Lead Equivalence (Pb_eq_)

The Pb equivalence (Pb_eq_) of the Sm_2_O_3_/HDPE, Eu_2_O_3_/HDPE, and Gd_2_O_3_/HDPE composites at photon energies of 0.06, 0.08, and 0.1 MeV, which are common X-ray energy ranges in general medical diagnostic and CT facilities, were determined using Equation (7):

(7) $$\mu_{\mathrm{Pb}}{\mathrm{Pb}}_{\mathrm{eq}}=\mu_{\mathrm{RO}/\mathrm{HDPE}}x$$

where µ_Pb_ (µ_RO/HDPE_) is the linear attenuation of pure Pb sheet (rare-earth oxide/HDPE composites), Pb_eq_ is the Pb equivalence (in mmPb), and x is the thickness of the sample (in mm), which was fixed at 10 mm in this work [48,49].

## 3. Results and Discussion

### 3.1. Mass Attenuation Coefficient (µ_m_)

**Figure 1:**
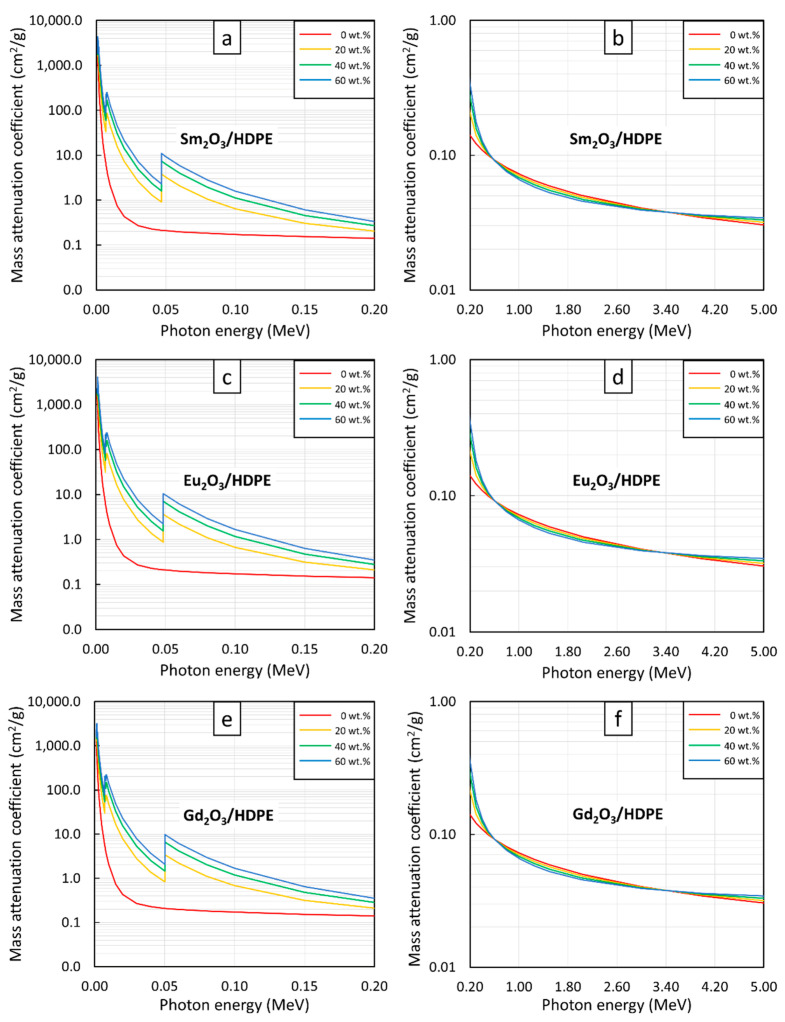
µ_m_ values of (**a**,**b**) Sm_2_O_3_/HDPE, (**c**,**d**) Eu_2_O_3_/HDPE, and (**e**,**f**) Gd_2_O_3_/HDPE composites with filler contents of 0, 20, 40, and 60 wt.%, determined at photon energies of (**a**,**c**,**e**) 0.001–0.2 MeV and (**b**,**d**,**f**) 0.2–5 MeV using XCOM.

The µ_m_ values for the Sm_2_O_3_/HDPE, Eu_2_O_3_/HDPE, and Gd_2_O_3_/HDPE composites at photon energies of 0.001–5 MeV and filler contents of 0, 20, 40, and 60 wt.% are shown in Figure 1 (raw data are available in supplementary; Tables S1–S4). The results indicated that the HDPE composites containing Sm_2_O_3_, Eu_2_O_3_, or Gd_2_O_3_ had notably higher µ_m_ values than the pristine HDPE composites at lower photon energies (0.001–0.2 MeV), as shown in Figure 1a,c,e. Furthermore, it was found that the µ_m_ values tended to increase with increasing filler contents but decreased with increasing photon energies. The shielding properties after the addition of rare-earth oxides were enhanced because the fillers could greatly increase the interaction probabilities between the incident photons and the composites from their relatively high Z values of Sm, Eu, and Gd, in Sm_2_O_3_, Eu_2_O_3_, or Gd_2_O_3_, respectively, through the process of photoelectric absorption, the cross section of which, a nuclear quantity representing the interaction probabilities of an element or a material with incident radiation, relates to the photon and material characteristics as shown in Equation (8):

(8) $$\sigma_{\mathrm{pe}}\propto \frac{Z^{n}}{{\left(h\nu \right)}^{3}}$$

where σ_pe_ is the photoelectric cross section, h is Planck’s constant, and ν is the frequency of the photon that directly relates to the energy (E=hν) [50].

However, in contrast to distinct µ_m_ variations for different filler contents at lower photon energies, the pristine HDPE and rare-earth oxide/HDPE composites had less pronounced differences in their µ_m_ values for all filler contents at photon energies greater than 0.5 MeV (Figure 1b,d,f). This was due to the rapid decrease in the photoelectric cross section with photon energies ($\sigma_{\mathrm{pe}}\propto \frac{1}{\nu^{3}}$) that greatly suppressed the roles of the added rare-earth oxides in photon attenuation through photoelectric absorption. It should be noted that at photon energies of 0.5–3.4 MeV, the pristine HDPE had slightly higher µ_m_ values than the rare-earth oxide/HDPE composites. This was due to the underlying principles of Compton scattering (a dominant attenuation mechanism for photons having energies in the range 0.5–3 MeV), which suggest that the Compton scattering cross section (σ_comp_) is inversely proportional to the electron density (n_e_) of the materials, as shown in Equation (9):

(9) $$\sigma_{\mathrm{comp}}\propto \frac{1}{n_{e}}$$

As a result, materials containing high contents of light elements such as the pristine HDPE would have higher σ_comp_ values and, subsequently, better photon attenuation abilities than those containing heavy elements, such as the rare-earth oxide/HDPE composites investigated in this work [43]. Nonetheless, at photon energies greater than 3.4 MeV, the µ_m_ values of the rare-earth oxide/HDPE composites began to regain their superior shielding properties, compared to the pristine HDPE. This was mainly due to the initiation of pair production at a photon energy of 1.022 MeV, with its cross section (σ_pp_) being directly proportional to the square of Z, as shown in Equation (10) [43,50]:

(10) $$\sigma_{\mathrm{pp}}\propto Z^{2}$$

and the value also tends to increase with increasing photon energies [51]. Thus, the added rare-earth oxides in the HDPE composites resumed their roles as active photon attenuators, resulting in enhanced µ_m_ values in the rare-earth oxide/HDPE composites at photon energies greater than 3.4 MeV.

**Figure 2:**
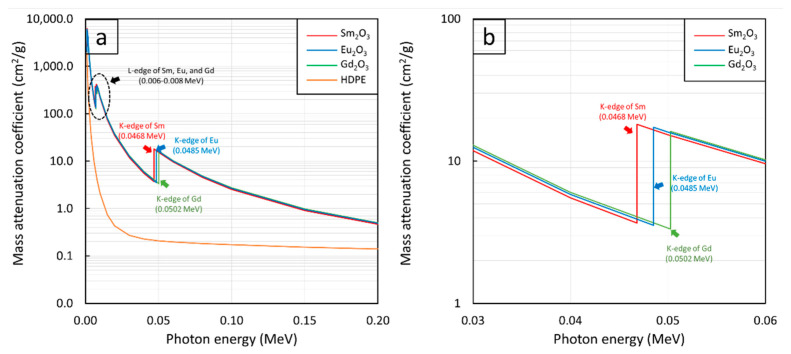
µ_m_ values of Sm_2_O_3_, Eu_2_O_3_, Gd_2_O_3_, and HDPE showing K-edge and L-edge behaviors of Sm, Eu, and Gd at photon energies of (**a**) 0.001–0.2 MeV and (**b**) 0.03–0.06 MeV.

Additionally, Figure 1a,c,e reveal uncharacteristically sharp increases in µ_m_ values at photon energies of 0.006–0.008 MeV and 0.04–0.05 MeV for all rare-earth oxide/HDPE composites. These phenomena were observed due to the K-edge and L-edge absorptions of Sm, Eu, and Gd atoms (Figure 2), for which the incident photon energies were just above the binding energy of the electron shells inside the atoms, resulting in immensely enhanced interaction probabilities between the incident photons and the materials through photoelectric absorption at these particular energies [52].

**Figure 3:**
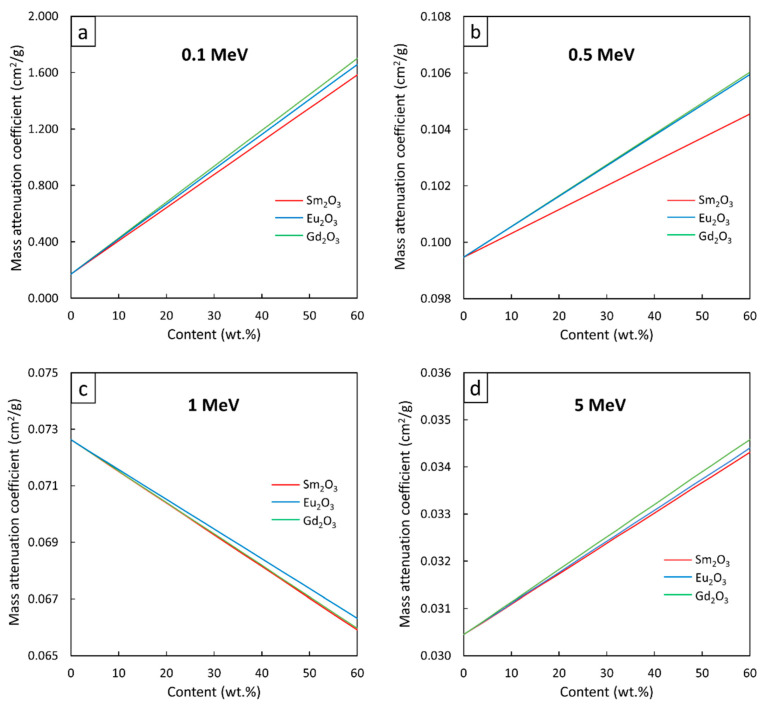
µ_m_ values of Sm_2_O_3_/HDPE, Eu_2_O_3_/HDPE, and Gd_2_O_3_/HDPE composites with filler contents varied from 0–60 wt.%, determined at photon energies of (**a**) 0.1 MeV, (**b**) 0.5 MeV, (**c**) 1 MeV, and (**d**) 5 MeV using XCOM.

The µ_m_ values for the Sm_2_O_3_/HDPE, Eu_2_O_3_/HDPE, and Gd_2_O_3_/HDPE composites with filler contents of 0–60 wt.%, determined at the photon energies of 0.1, 0.5, 1, and 5 MeV, are shown in Figure 3, with strong linear correlations evidenced between the filler contents and the µ_m_ values at all investigated energies. In particular, as shown in Figure 3a,b,d, there were positive correlations between the µ_m_ values and filler contents, mainly due to the potent roles of the Sm_2_O_3_, Eu_2_O_3_, or Gd_2_O_3_ particles in photon attenuation through the dominant photoelectric absorption at 0.1 and 0.5 MeV and the pair production at 5 MeV (the two mechanisms were highly dependent on the Z value). This positive correlation implied that higher filler contents would result in more available rare-earth elements to interact with the incident photons, resulting in enhanced interaction probabilities and, consequently, improved shielding abilities. The schemes showing effects of radiation-protective fillers and their contents on photon attenuation can be viewed elsewhere [43,53].

On the other hand, Figure 3c shows negative correlations between the variables, as higher filler contents led to lower µ_m_ values. This trend in behavior was because the pristine HDPE, which is a hydrogen-rich material, could interact with the incident photons through Compton scattering, a dominant photon interaction at 1 MeV, at higher probabilities than those of the rare-earth oxide/HDPE composites, which had less light-element contents due to the dilution effects from the added rare-earth oxides. This phenomenon could be mathematically explained using Equation (7) as σ_comp_ is inversely proportional to the electron densities of the composites.

To determine the µ_m_ values for all filler contents at photon energies of 0.1, 0.5, 1, and 5 MeV (Figure 3), linear mathematical equations in the form of µ_m_ = Ax + B (Equation (1)) were determined using a trendline function in Microsoft Excel. The results, as shown in Table 2, indicated that the Gd_2_O_3_/HDPE composites had the strongest correlations between µ_m_ values and filler contents among all the investigated rare-earth oxide/HDPE composites, as seen by the highest slopes (coefficient A) for all photon energies. These were due to Gd having the highest Z value (Z = 64) compared to Sm (Z = 62) and Eu (Z = 63), which resulted in more chances of interaction between the incident photons and the materials and, hence, a greater effect of the filler on enhancing the shielding ability. Notably, both coefficients A and B for the 0.1-MeV photon attenuations were higher than those at higher energies for all composites. This was due to the photoelectric absorption, which is a dominant interaction at 0.1 MeV and heavily reliant on the Z values of the composites (Equation (6)) than those from Compton scattering (Equation (7)) and pair production (Equation (8)), leading to more pronounced changes in the µ_m_ values as more filler was added to each HDPE composite.

To validate the results from XCOM in this work, other available programs, namely Phy-X/PSD and PHITS, were used to calculate the µ_m_ values of the rare-earth oxide/HDPE composites with filler contents of 20, 40, and 60 wt.% at photon energies of 0.1, 0.5, 1, and 5 MeV. The results of the comparisons, as well as the percentage of differences (Difference (%)) between µ_m_ values obtained from each software package (Equation (2)) are shown in Table 3, which indicated that the range for Difference (%) between XCOM vs. Phy-X/PSD and XCOM vs. PHITS were 0.00–0.05% with the average being 0.02% and 0.02–1.24% with the average being 0.56%, respectively. These comparisons clearly showed that the determined results from XCOM were in very good agreement with those using the other software packages; hence, they could be reliably used in later determinations of µ, HVL, and Pb_eq_. It should be noted that the differences in results obtained from the three methods could have been due to several factors, including possible deviations in the cross-section databases, use of incoherent/coherent scattering for the calculations, or the mathematical corrections used for the calculation of µ_m_ [44,46,47].

### 3.2. Linear Attenuation Coefficients (µ) and Half Value Layer (HVL)

The densities for each filler content were measured to determine the µ and HVL values of the rare-earth oxide/HDPE composites. The results of the density calculations based on Equation (5) are shown in Table 4, which suggested that the densities of the rare-earth oxide/HDPE composites generally increased with filler contents due to the high densities of the rare-earth oxide fillers. It was notable that the Sm_2_O_3_/HDPE composites had slightly higher densities than the Eu_2_O_3_/HDPE and Gd_2_O_3_/HDPE composites at the same filler content due to higher density of Sm_2_O_3_ than for Eu_2_O_3_ and Gd_2_O_3_ (Table 1).

**Figure 4:**
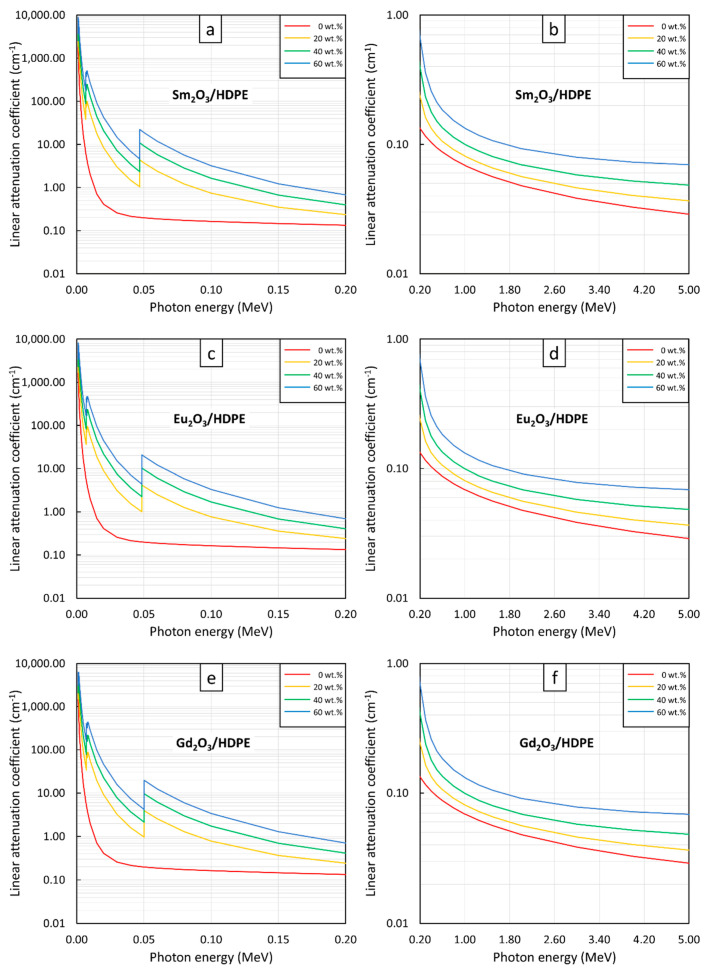
µ values of (**a**,**b**) Sm_2_O_3_/HDPE, (**c**,**d**) Eu_2_O_3_/HDPE, and (**e**,**f**) Gd_2_O_3_/HDPE composites with filler contents of 0, 20, 40, and 60 wt.%, determined at photon energies of (**a**,**c**,**e**) 0.001–0.2 MeV and (**b**,**d**,**f**) 0.2–5 MeV using XCOM.

**Figure 5:**
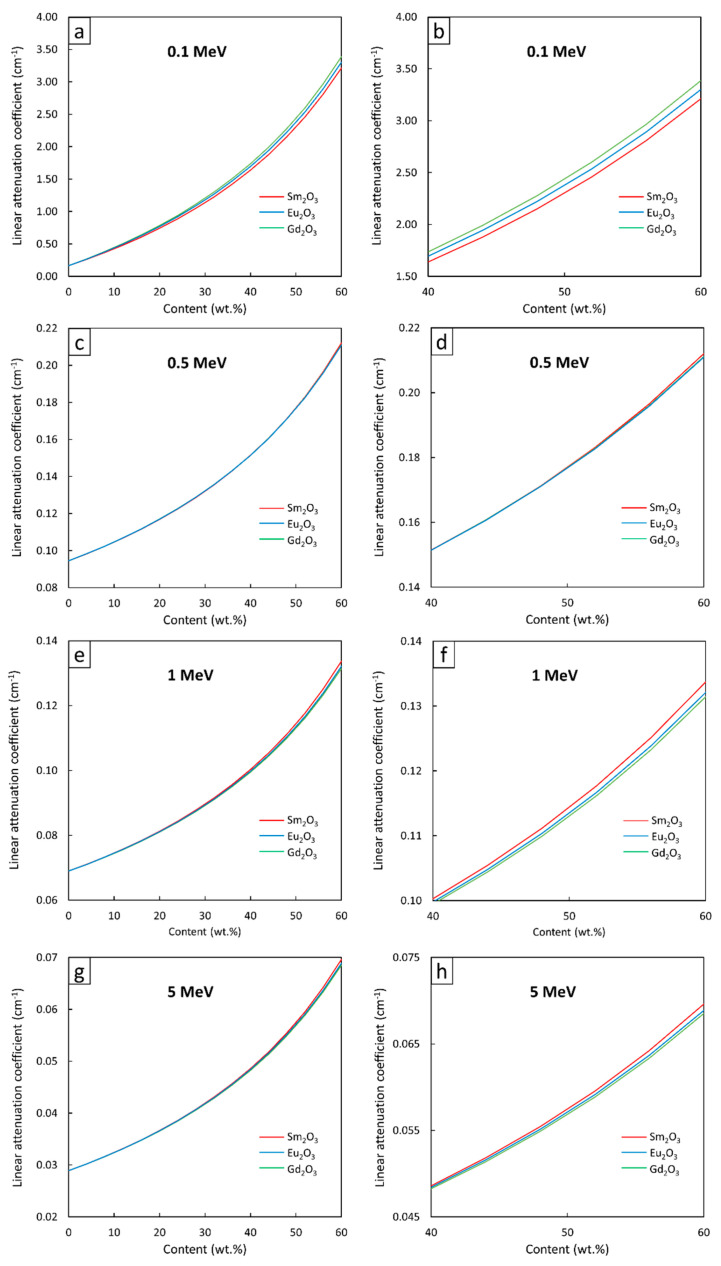
µ values of Sm_2_O_3_/HDPE, Eu_2_O_3_/HDPE, and Gd_2_O_3_/HDPE composites using XCOM, with filler contents varied from (**a**,**c**,**e**,**g**) 0–60 wt.%, and (**b**,**d**,**f**,**h**) 40–60 wt.%, determined at photon energies of (**a**,**b**) 0.1 MeV, (**c**,**d**) 0.5 MeV, (**e**,**f**) 1 MeV, and (**g**,**h**) 5 MeV.

**Figure 6:**
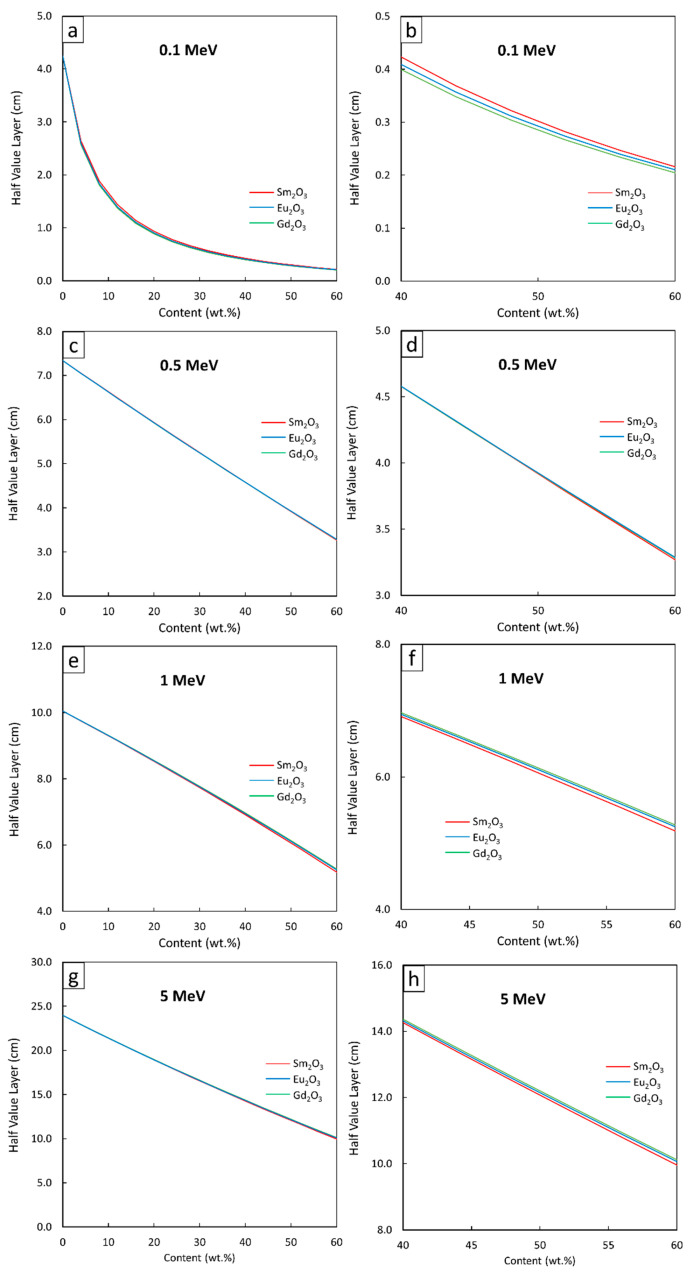
HVL values of Sm_2_O_3_/HDPE, Eu_2_O_3_/HDPE, and Gd_2_O_3_/HDPE composites using XCOM, with filler contents varied from (**a**,**c**,**e**,**g**) 0–60 wt.%, and (**b**,**d**,**f**,**h**) 40–60 wt.%, determined at photon energies of (**a**,**b**) 0.1 MeV, (**c**,**d**) 0.5 MeV, (**e**,**f**) 1 MeV, and (**g**,**h**) 5 MeV.

Figure 4, Figure 5 and Figure 6 show the µ and HVL values of the Sm_2_O_3_/HDPE, Eu_2_O_3_/HDPE, and Gd_2_O_3_/HDPE composites with varying fillers contents and photon energies. The results in Figure 4 indicated that the µ values for all composites had similar trends, namely increasing with filler content but decreasing with photon energy. These results agreed with the behavior of µ_m_ (Figure 1) due to the abilities of the rare-earth oxides to enhance the interaction probabilities between the incident photons and the materials. Furthermore, Figure 5 and Figure 6 suggest that the effects of the additional filler contents on the µ and HVL values for all composites were more pronounced than those observed in µ_m_, as seen by the exponential correlations between the µ (HVL) values and the filler contents (the relationships between µ_m_ and filler contents were linearly dependent (Figure 3)). This stronger dependence of the µ and HVL values on the filler content was mainly due to the relatively high densities of Sm_2_O_3_, Eu_2_O_3_, and Gd_2_O_3_ that increased the densities of the composites at higher filler contents, which subsequently amplified the µ values according to Equations (3) and (4).

Similar to µ_m_, mathematical correlations between µ (HVL) values and the filler contents were determined at photon energies of 0.1, 0.5, 1, and 5 MeV and the results are shown in Table 5 and Table 6, respectively. It should be noted that the absolute values of coefficient B, which indicate the strength of the correlation between µ (HVL) and filler contents, determined at 0.1-MeV photons, were higher than those of 0.5-, 1-, and 5-MeV photons. This was because the additional rare-earth oxides could greatly increase the photon interactions of the composites through the most effective photoelectric absorption at lower photon energies, leading to more pronounced enhancement in the µ and HVL values.

### 3.3. Lead Equivalence (Pb_eq_)

To determine the useability of the developed composites for actual applications, especially in medical applications, the Pb equivalence (Pb_eq_) values were determined for all composites at photon energies of 0.06, 0.08, and 0.1 MeV and filler contents of 20, 40, and 60 wt.%, and are shown in Table 7. The results indicated that the Pb_eq_ values increased with increasing filler content, which was consistent with the behaviors of µ_m_ and µ, with the highest Pb_eq_ values being 2.17, 2.17, and 0.54 mmPb at the photon energies of 0.06, 0.08, and 0.1 MeV, respectively, achieved in 60 wt.%-Gd_2_O_3_/HDPE composites. Although the requirement of minimum Pb_eq_ for X-ray shielding materials varied, depending on the photon energy, equipment, and applications, most of the general medical diagnostic X-ray and CT facilities require Pb_eq_ values of at least 1 mmPb at 100 kVp (0.06–0.08 MeV) and 1.5 mmPb at 120 kVp (~0.08 MeV), respectively [54]. Hence, Table 7 suggests that the HDPE composites with at least 40 wt.% and 50 wt.% (interpolated from the composites with 40 wt.% and 60 wt.%) were the recommended formulations for applications in general medical diagnostic X-ray and CT rooms, respectively. It should be noted that the actual photon energies inside the facilities were distributed as a spectrum, with their average energies varying depending on the type, design, and manufacturer of the X-ray equipment and facilities.

### 3.4. Comparative µ_m_, µ, and HVL of Rare-Earth Oxide/HDPE and Other Common Pb-Free Composites

**Figure 7:**
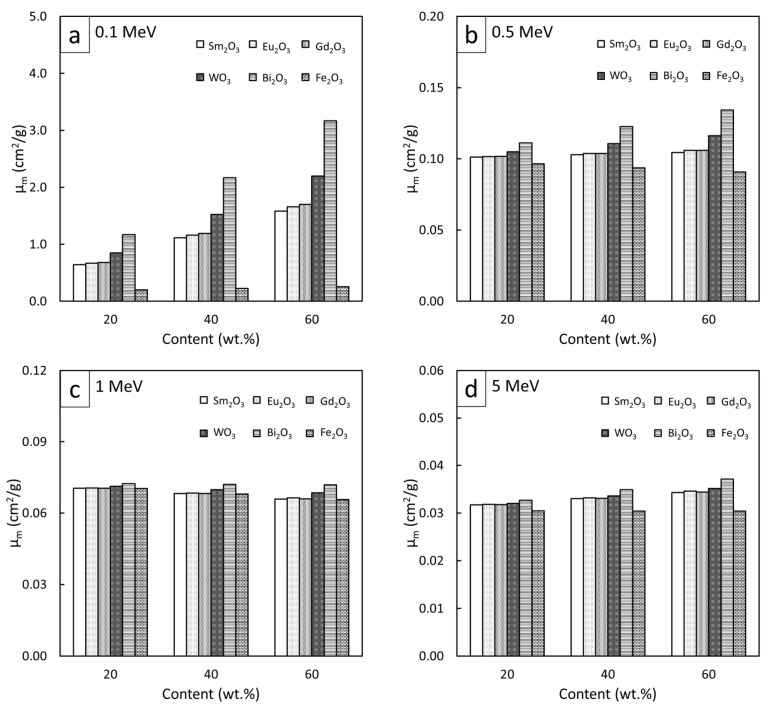
Comparative µ_m_ values of Sm_2_O_3_/HDPE, Eu_2_O_3_/HDPE, and Gd_2_O_3_/HDPE composites with other common Pb-free HDPE composites (Bi_2_O_3_/HDPE, WO_3_/HDPE, and Fe_2_O_3_/HDPE) at filler contents of 20, 40, and 60 wt.% and photon energies of (**a**) 0.1 MeV, (**b**) 0.5 MeV, (**c**) 1 MeV, and (**d**) 5 MeV.

**Figure 8:**
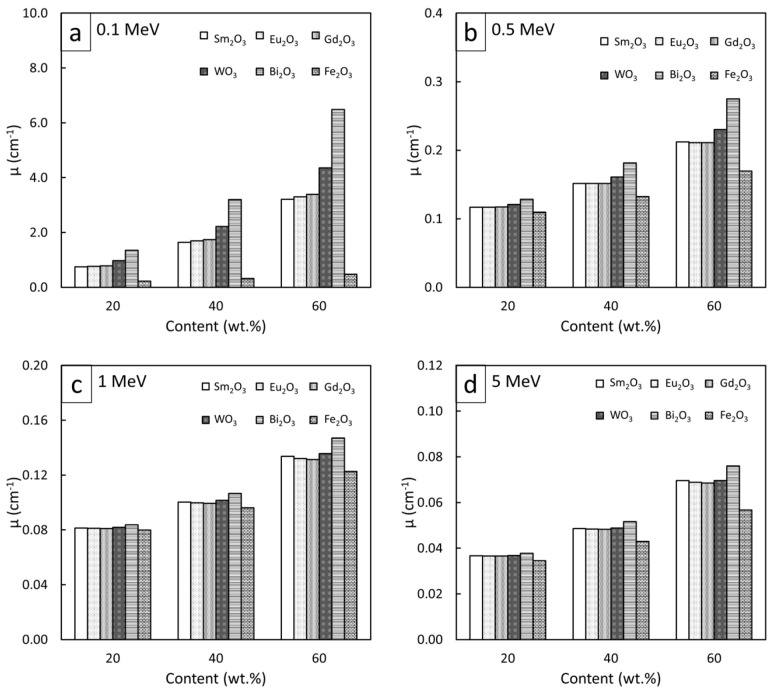
Comparative µ values of Sm_2_O_3_/HDPE, Eu_2_O_3_/HDPE, and Gd_2_O_3_/HDPE composites with other common Pb-free HDPE composites (Bi_2_O_3_/HDPE, WO_3_/HDPE, and Fe_2_O_3_/HDPE) at filler contents of 20, 40, and 60 wt.% and photon energies of (**a**) 0.1 MeV, (**b**) 0.5 MeV, (**c**) 1 MeV, and (**d**) 5 MeV.

**Figure 9:**
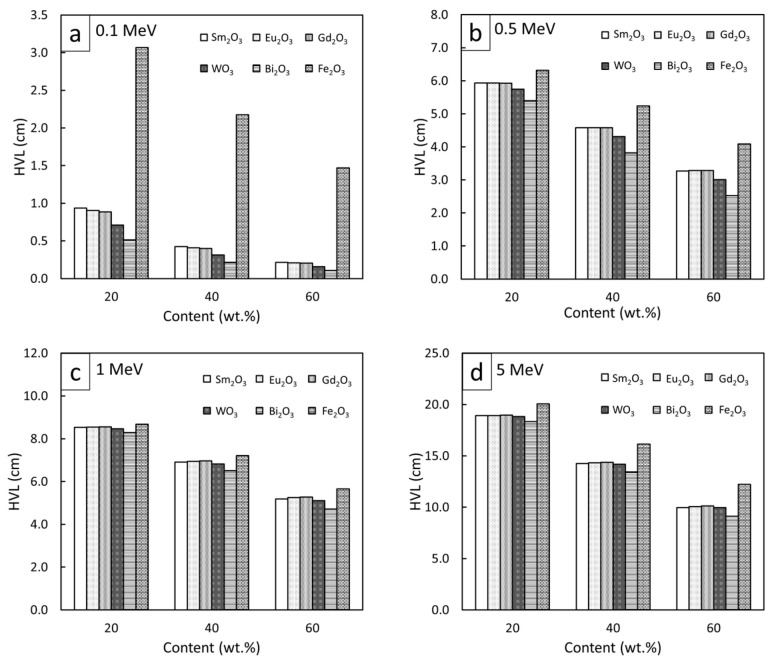
Comparative HVL values of Sm_2_O_3_/HDPE, Eu_2_O_3_/HDPE, and Gd_2_O_3_/HDPE composites with other common Pb-free HDPE composites (Bi_2_O_3_/HDPE, WO_3_/HDPE, and Fe_2_O_3_/HDPE) at filler contents of 20, 40, and 60 wt.% and photon energies of (**a**) 0.1 MeV, (**b**) 0.5 MeV, (**c**) 1 MeV, and (**d**) 5 MeV.

To compare the high-energy photon shielding capabilities of the Sm_2_O_3_/HDPE, Eu_2_O_3_/HDPE, and Gd_2_O_3_/HDPE composites with other common Pb-free HDPE composites (Bi_2_O_3_/HDPE, WO_3_/HDPE, and Fe_2_O_3_/HDPE), the µ_m_, µ, and HVL values for all the composites of interest were determined and are shown in Figure 7, Figure 8 and Figure 9, respectively (raw data are available in supplementary; Table S5). The results revealed that Bi_2_O_3_/HDPE composites had the highest shielding abilities among all the composites as seen by their highest values of µ_m_ and µ, and their lowest values of HVL. This was because the Bi atoms and Bi_2_O_3_ in the Bi_2_O_3_/HDPE composites have a higher Z (Z = 83) and density (8.90 g/cm^3^) than the other fillers, leading to higher chances of interactions with incident photons. In contrast, the Fe_2_O_3_/HDPE composites had the lowest shielding properties, mainly due to the low Z (Z = 26) of the Fe atoms and the lowest density (ρ = 5.24 g/cm^3^) of Fe_2_O_3_ that resulted in lower interaction probabilities, and consequently, inferior overall shielding properties. Notably, the rare-earth oxides/HDPE and WO_3_/HDPE composites attenuated high-energy photons with comparable shielding capabilities. For example, the µ values of the WO_3_/HDPE composites were higher than those of the Gd_2_O_3_/HDPE composites by 24%, 8%, 3%, and 1% at photon energies of 0.1, 0.5, 1, and 5 MeV, respectively, implying promising utilization of the rare-earth oxide/HDPE composites as Pb-free shielding materials, especially where the photon energy is greater than 0.5 MeV.

Another interesting advantage of the rare-earth oxide/HDPE composites over other common Pb-free shielding materials was the simultaneous ability of the former to competently attenuate both high-energy photons and thermal neutrons because Sm, Eu, and Gd have relatively high Z values (making them suitable for photon attenuation) and excellent σ_abs_ values (making them suitable for thermal neutron absorption). Furthermore Bi, W, and Fe have considerably smaller σ_abs_ values than Sm, Eu, and Gd; hence, they are not suitable for thermal neutron attenuation [55,56]. This dual shielding property of the rare-earth oxide/HDPE composites would enable the developed materials to be used in neutron–photon-mixed environments, such as near nuclear reactors or ion accelerators, which could subsequently reduce the required amount of individual shielding material, as well as allowing a simplified design for the shielding setup [32].

In addition to the comparison between the results obtained from this work and those from common HDPE composites, our results were also compared with previously reported high-energy photon shielding properties of glassy alloys containing different types and contents of rare-earth elements, namely Gd, Tb, Dy, Ho, Er, and Tm. The results of the comparison are shown in Table 8, which indicated that the rare-earth oxide/HDPE composites investigated in this work exhibited lower overall photon shielding properties than glassy alloys, as seen by lower µ_m_ and µ values and higher HVL values at the same photon energy. These results were mainly observed due to higher weight fractions of rare-earth elements in glassy alloys (total weight fractions of rare-earth elements in the alloys were approximately 0.8–0.85) than those in HDPE composites (weight fractions of Sm, Eu, and Gd were approximately 0.5) as well as their much higher densities of glassy alloys (ρ = 6.898–7.68 g/cm^3^) in comparison to HDPE composites (the highest ρ was approximately 2 g/cm^3^ for those with 60 wt.% rare-earth oxides), which resulted in higher photon interaction probabilities and overall shielding abilities in glassy alloys. Nonetheless, the rare-earth oxide/HDPE composites in this work could still be useful in actual applications, especially for medical purposes, for which the HDPE composites offer not only sufficient photon attenuation abilities for the safety of the users but also their lighter weight and high strength, which enabled the materials to be used as structural parts and transporting casks.

## 4. Conclusions

This work determined the theoretical high-energy photon shielding properties (µ_m_, µ, HVL, and Pb_eq_) for Sm_2_O_3_/HDPE, Eu_2_O_3_/HDPE, and Gd_2_O_3_/HDPE composites with filler contents in the range 0–60 wt.% and photon energies in the range 0.001–5 MeV for the development of Pb-free materials to shield against X-rays and gamma rays with exceptional strength and rigidity. The XCOM simulation software was used in this work and the results were verified with other programs, namely Phy-X/PSD and PHITS, which showed very good agreement among the three methods. The results showed that the overall high-energy photon shielding properties and densities of the composites increased with the addition of rare-earth oxide fillers, as seen by the increases (decreases) in µ_m_, µ, and ρ (HVL), but these properties were lowered with increasing photon energies. Furthermore, the Pb_eq_ determination indicated that the rare-earth oxide/HDPE composites with 40 wt.% and 50 wt.% filler contents were suitable for use in general diagnostic X-ray and CT facilities as their Pb_eq_ values at these recommended contents were higher than 1 mmPb and 1.5 mmPb, respectively. Compared to other Pb-free shielding materials, namely HDPE composites and glassy alloys, the developed rare-earth oxide/HDPE composites could attenuate incident photons with comparable efficiencies to some of the common materials, for example, to WO_3_/HDPE composites, especially at photon energies greater than 0.5 MeV, implying the promising potential of utilizing rare-earth oxide as alternative radiation protective fillers. Additionally, the rare-earth oxide/HDPE composites had advantages compared to other materials due to these composites with the dual capabilities of efficient high-energy photon and thermal neutron attenuations.

## Figures and Tables

**Table 1:** Individual density of a pristine HDPE, rare-earth oxides, metal oxides, and Pb used for determination of densities for HDPE composites [22,35].

Matrix/Compound	Density (g/cm^3^)
HDPE	0.95
Sm_2_O_3_	8.35
Eu_2_O_3_	7.41
Gd_2_O_3_	7.40
WO_3_	7.16
Bi_2_O_3_	8.90
Fe_2_O_3_	5.24
Pb	11.35

**Table 2:** Mathematical coefficients (A and B) for µ_m_ in form µ_m_ = Ax + B (Equation (1)) from Figure 3.

Photon Energy (MeV)	Sm_2_O_3_		Eu_2_O_3_		Gd_2_O_3_	
	A	B	A	B	A	B
0.1	0.0235	0.172	0.0248	0.172	0.0255	0.172
0.5	0.85 × 10^−4^	0.099	1.08 × 10^−4^	0.099	1.09 × 10^−4^	0.099
1.0	−1.12 × 10^−4^	0.073	−1.05 × 10^−4^	0.073	−1.11 × 10^−4^	0.073
5.0	6.44 × 10^−5^	0.030	6.58 × 10^−5^	0.030	6.89 × 10^−5^	0.030

**Table 3:** Comparative µ_m_ values obtained from XCOM and other programs (Phy-X/PSD and PHITS) for HDPE composites containing rare-earth oxides at various photon energies.

Filler	Photon Energy (MeV)	Content (wt.%)	µ_m_ (cm^2^/g)			Difference (%)	
			XCOM	Phys-X (PSD)	PHITS	XCOM vs. Phys-X (PSD)	XCOM vs. PHITS
Sm_2_O_3_	0.1	20	0.64220	0.64223	0.64905	0.01	1.06
		40	1.11300	1.11255	1.11783	0.04	0.43
		60	1.58300	1.58287	1.58001	0.01	0.18
	0.5	20	0.10120	0.10116	0.10127	0.04	0.06
		40	0.10290	0.10285	0.10331	0.05	0.39
		60	0.10450	0.10454	0.10458	0.04	0.07
	1.0	20	0.07039	0.07039	0.07104	0.01	0.92
		40	0.06815	0.06815	0.06841	0.01	0.38
		60	0.06591	0.06591	0.06596	0.01	0.07
	5.0	20	0.03173	0.03173	0.03194	0.01	0.66
		40	0.03302	0.03302	0.03338	0.00	1.09
		60	0.03431	0.03431	0.03466	0.01	1.02
Eu_2_O_3_	0.1	20	0.66690	0.66691	0.67318	0.00	0.94
		40	1.16200	1.16191	1.16680	0.01	0.41
		60	1.65700	1.65691	1.65340	0.01	0.21
	0.5	20	0.10160	0.10163	0.10176	0.03	0.15
		40	0.10380	0.10379	0.10331	0.01	0.47
		60	0.10590	0.10595	0.10458	0.05	1.24
	1.0	20	0.07052	0.07052	0.07121	0.00	0.97
		40	0.06842	0.06842	0.06868	0.00	0.38
		60	0.06632	0.06632	0.06639	0.01	0.10
	5.0	20	0.03183	0.03183	0.03204	0.01	0.65
		40	0.03320	0.03320	0.03358	0.01	1.14
		60	0.03458	0.03458	0.03491	0.01	0.95
Gd_2_O_3_	0.1	20	0.68120	0.68118	0.68734	0.00	0.90
		40	1.19000	1.19045	1.19608	0.04	0.51
		60	1.70000	1.69971	1.69741	0.02	0.15
	0.5	20	0.10170	0.10166	0.10178	0.04	0.07
		40	0.10380	0.10384	0.10427	0.04	0.45
		60	0.10600	0.10603	0.10603	0.03	0.02
	1.0	20	0.07041	0.07041	0.07107	0.00	0.93
		40	0.06819	0.06819	0.06844	0.00	0.36
		60	0.06597	0.06597	0.06601	0.00	0.06
	5.0	20	0.03176	0.03176	0.03197	0.01	0.66
		40	0.03308	0.03308	0.03344	0.00	1.08
		60	0.03440	0.03440	0.03472	0.01	0.93

**Table 4:** Densities of Sm_2_O_3_/HDPE, Eu_2_O_3_/HDPE, and Gd_2_O_3_/HDPE composites with filler contents varying from 0 to 60 wt.% (in 4 wt.% increments), calculated using Equation (5).

Content (wt.%)	Density (g/cm^3^)		
	Sm_2_O_3_	Eu_2_O_3_	Gd_2_O_3_
0	0.95	0.95	0.95
4	0.98	0.98	0.98
8	1.02	1.02	1.02
12	1.06	1.06	1.06
16	1.11	1.10	1.10
20	1.15	1.15	1.15
24	1.21	1.20	1.20
28	1.26	1.26	1.26
32	1.33	1.32	1.32
36	1.40	1.38	1.38
40	1.47	1.46	1.46
44	1.56	1.54	1.54
48	1.65	1.63	1.63
52	1.76	1.74	1.74
56	1.89	1.86	1.86
60	2.03	1.99	1.99

**Table 5:** Mathematical constants (A and B) of µ in the form µ = Ae^Bx^ (Equation (6)) from Figure 5.

Photon Energy (MeV)	Sm_2_O_3_		Eu_2_O_3_		Gd_2_O_3_	
	A	B	A	B	A	B
0.1	0.2534	0.0453	0.2591	0.0456	0.2622	0.0459
0.5	0.0909	0.0133	0.0911	0.0132	0.0911	0.0132
1	0.0665	0.0105	0.0664	0.0108	0.0665	0.0106
5	0.0279	0.0144	0.0279	0.0143	0.0279	0.0142

**Table 6:** Mathematical constants (A and B) of HVL in the form HVL = Ae^Bx^ (Equation (6)) from Figure 6.

Photon Energy (MeV)	Sm_2_O_3_		Eu_2_O_3_		Gd_2_O_3_	
	A	B	A	B	A	B
0.1	2.735	−0.0453	2.675	−0.0456	2.644	−0.0459
0.5	7.622	−0.0133	7.608	−0.0132	7.609	−0.0132
1	10.42	−0.0108	10.42	−0.0106	10.42	−0.0106
5	24.88	−0.0144	24.83	−0.0143	24.83	−0.0142

**Table 7:** Pb_eq_ of Sm_2_O_3_/HDPE, Eu_2_O_3_/HDPE, and Gd_2_O_3_/HDPE composites with filler contents of 0, 20, 40, and 60 wt.%, determined at photon energies of 0.06, 0.08, and 0.1 MeV using XCOM.

Photon Energy (MeV)	Filler	Lead Equivalence (mmPb)		
		20 wt.%	40 wt.%	60 wt.%
0.06	Sm_2_O_3_	0.42	1.02	2.07
	Eu_2_O_3_	0.44	1.05	2.13
	Gd_2_O_3_	0.45	1.08	2.17
0.08	Sm_2_O_3_	0.44	1.03	2.06
	Eu_2_O_3_	0.46	1.06	2.11
	Gd_2_O_3_	0.47	1.09	2.17
0.1	Sm_2_O_3_	0.11	0.26	0.51
	Eu_2_O_3_	0.12	0.27	0.52
	Gd_2_O_3_	0.12	0.28	0.54

**Table 8:** Comparative µ_m_, µ, and HVL values of Sm_2_O_3_/HDPE, Eu_2_O_3_/HDPE, and Gd_2_O_3_/HDPE composites with glassy alloys containing different types and contents of rare-earth elements (Gd, Tb, Dy, Ho, Er, and Tm) at photon energies of 0.1 and 5 MeV.

Photon Energy (MeV)	Material	Rare-Earth Element (Weight Fraction in the Material)	µ_m_ (cm^2^/g)	µ (cm^−1^)	HVL (cm)	Reference
0.1	HDPE	Sm (0.5174)	1.583	3.213	0.216	This work
	HDPE	Eu (0.5182)	1.657	3.297	0.210	
	HDPE	Gd (0.5206)	1.700	3.383	0.205	
	Glassy alloys	Gd (0.3911), Tb (0.3952)	2.451	16.906	0.041	[30]
	Glassy alloys	Gd (0.3877), Dy (0.4006)	2.498	17.429	0.039	
	Glassy alloys	Gd (0.3853), Ho (0.4041)	2.554	17.980	0.039	
	Glassy alloys	Tb (0.2766), Dy (0.2828), Er (0.2911)	2.821	21.050	0.033	
	Glassy alloys	Dy (0.2780), Er (0.2861), Tm (0.2890)	2.973	22.833	0.030	
	Glassy alloys	Gd (0.2786), Tb (0.2816), Dy (0.2879)	2.675	19.982	0.035	
	Glassy alloys	Gd (0.2785), Tb (0.2815), Dy (0.2878)	2.669	19.916	0.035	
5.0	HDPE	Sm (0.5174)	0.034	0.069	10.046	This work
	HDPE	Eu (0.5182)	0.035	0.070	9.902	
	HDPE	Gd (0.5206)	0.034	0.068	10.193	
	Glassy alloys	Gd (0.3911), Tb (0.3952)	0.037	0.255	2.718	[30]
	Glassy alloys	Gd (0.3877), Dy (0.4006)	0.037	0.258	2.687	
	Glassy alloys	Gd (0.3853), Ho (0.4041)	0.037	0.260	2.666	
	Glassy alloys	Tb (0.2766), Dy (0.2828), Er (0.2911)	0.038	0.284	2.441	
	Glassy alloys	Dy (0.2780), Er (0.2861), Tm (0.2890)	0.038	0.292	2.374	
	Glassy alloys	Gd (0.2786), Tb (0.2816), Dy (0.2879)	0.038	0.284	2.441	
	Glassy alloys	Gd (0.2785), Tb (0.2815), Dy (0.2878)	0.038	0.284	2.441	

## Supplementary Materials

The following are available online at https://www.mdpi.com/article/10.3390/polym13121930/s1, Table S1: Mass attenuation coefficients (µ_m_) of HDPE composites at photon energies of 0.001–5 MeV, Table S2: Mass attenuation coefficients (µ_m_) of Sm_2_O_3_/HDPE composites at filler contents of 20, 40, and 60 wt.% and photon energies of 0.001–5 MeV, Table S3: Mass attenuation coefficients (µ_m_) of Eu_2_O_3_/HDPE composites at filler contents of 20, 40, and 60 wt.% and photon energies of 0.001–5 MeV, Table S4: Mass attenuation coefficients (µ_m_) of Gd_2_O_3_/HDPE composites at filler contents of 20, 40, and 60 wt.% and photon energies of 0.001–5 MeV, Table S5: Comparative µ_m_, µ, and HVL values of Sm_2_O_3_/HDPE, Eu_2_O_3_/HDPE, and Gd_2_O_3_/HDPE composites with other common Pb-free HDPE composites (Bi_2_O_3_/HDPE, WO_3_/HDPE, and Fe_2_O_3_/HDPE) at filler contents of 20, 40, and 60 wt.% and photon energies of 0.1, 0.5, 1, and 5 MeV.
