# Supplementary material for: Rare-Earth Oxides as Alternative High-Energy Photon Protective Fillers in HDPE Composites: Theoretical Aspects
Source: Polymers (Basel). 2021 Jun 10;13(12):1930. doi: 10.3390/polym13121930 (PMC8230413; doi:10.3390/polym13121930)
Supplement: Supplementary file 1 [file polymers-13-01930-s001.zip › polymers-1223584-supplementary.pdf]

Supporting Information

Table 1. Mass attenuation coefficients (μ_m) of HDPE composites at photon energies of 0.001–5 MeV.

Photon energy (MeV).	μ_m (cm^2/g)
1.00E-03	1.89E+03
1.04E-03	1.70E+03
1.08E-03	1.52E+03
1.08E-03	1.52E+03
1.09E-03	1.48E+03
1.11E-03	1.43E+03
1.11E-03	1.43E+03
1.25E-03	1.00E+03
1.42E-03	7.03E+02
1.42E-03	7.03E+02
1.50E-03	6.00E+02
1.54E-03	5.56E+02
1.54E-03	5.56E+02
1.63E-03	4.73E+02
1.72E-03	4.02E+02
1.72E-03	4.02E+02
2.00E-03	2.59E+02
3.00E-03	7.74E+01
4.00E-03	3.24E+01
5.00E-03	1.64E+01
6.00E-03	9.43E+00
6.72E-03	6.70E+00
6.72E-03	6.70E+00
7.01E-03	5.90E+00
7.31E-03	5.19E+00
7.31E-03	5.19E+00
7.52E-03	4.77E+00
7.74E-03	4.39E+00
7.74E-03	4.39E+00
8.00E-03	3.98E+00
1.00E-02	2.09E+00
1.50E-02	7.46E-01
2.00E-02	4.32E-01
3.00E-02	2.71E-01
4.00E-02	2.28E-01
4.68E-02	2.13E-01
4.68E-02	2.13E-01
5.00E-02	2.08E-01
6.00E-02	1.97E-01
8.00E-02	1.82E-01
1.00E-01	1.72E-01
1.50E-01	1.53E-01
2.00E-01	1.40E-01
3.00E-01	1.22E-01
4.00E-01	1.09E-01
5.00E-01	9.95E-02
6.00E-01	9.20E-02
8.00E-01	8.08E-02
1.00E+00	7.26E-02
1.02E+00	7.19E-02
1.25E+00	6.50E-02
1.50E+00	5.91E-02
2.00E+00	5.06E-02

2.04E+00	5.01E-02
3.00E+00	4.05E-02
4.00E+00	3.44E-02
5.00E+00	3.05E-02

Table 2. Mass attenuation coefficients (μ_m) of Sm_2O_3/HDPE composites at filler contents of 20, 40, and 60 wt.% and photon energies of 0.001–5 MeV.

Photon energy (MeV)	Content (wt.%)		
	20	40	60
1.00E-03	2.01E+03	2.12E+03	2.23E+03
1.04E-03	1.81E+03	1.92E+03	2.04E+03
1.08E-03	1.64E+03	1.75E+03	1.86E+03
1.08E-03	1.76E+03	1.99E+03	2.23E+03
1.09E-03	1.93E+03	2.38E+03	2.83E+03
1.11E-03	2.20E+03	2.97E+03	3.74E+03
1.11E-03	2.41E+03	3.39E+03	4.38E+03
1.25E-03	1.90E+03	2.80E+03	3.70E+03
1.42E-03	1.52E+03	2.34E+03	3.15E+03
1.42E-03	1.66E+03	2.62E+03	3.57E+03
1.50E-03	1.45E+03	2.29E+03	3.14E+03
1.54E-03	1.36E+03	2.15E+03	2.95E+03
1.54E-03	1.41E+03	2.26E+03	3.11E+03
1.63E-03	1.23E+03	1.99E+03	2.74E+03
1.72E-03	1.07E+03	1.75E+03	2.42E+03
1.72E-03	1.11E+03	1.81E+03	2.51E+03
2.00E-03	7.65E+02	1.27E+03	1.78E+03
3.00E-03	2.74E+02	4.70E+02	6.67E+02
4.00E-03	1.30E+02	2.27E+02	3.24E+02
5.00E-03	7.24E+01	1.28E+02	1.84E+02
6.00E-03	4.48E+01	8.01E+01	1.16E+02
6.72E-03	3.33E+01	5.99E+01	8.65E+01
6.72E-03	8.07E+01	1.55E+02	2.29E+02
7.01E-03	7.23E+01	1.39E+02	2.05E+02
7.31E-03	6.49E+01	1.25E+02	1.84E+02
7.31E-03	8.66E+01	1.68E+02	2.49E+02
7.52E-03	8.08E+01	1.57E+02	2.33E+02
7.74E-03	7.53E+01	1.46E+02	2.17E+02
7.74E-03	8.63E+01	1.68E+02	2.50E+02
8.00E-03	7.94E+01	1.55E+02	2.30E+02
1.00E-02	4.49E+01	8.78E+01	1.31E+02
1.50E-02	1.55E+01	3.03E+01	4.51E+01
2.00E-02	7.31E+00	1.42E+01	2.11E+01
3.00E-02	2.58E+00	4.89E+00	7.20E+00
4.00E-02	1.29E+00	2.35E+00	3.40E+00
4.68E-02	9.03E-01	1.59E+00	2.28E+00
4.68E-02	3.80E+00	7.38E+00	1.10E+01
5.00E-02	3.23E+00	6.26E+00	9.28E+00
6.00E-02	2.07E+00	3.95E+00	5.82E+00
8.00E-02	1.05E+00	1.92E+00	2.78E+00
1.00E-01	6.42E-01	1.11E+00	1.58E+00
1.50E-01	3.04E-01	4.54E-01	6.04E-01
2.00E-01	2.05E-01	2.70E-01	3.35E-01
3.00E-01	1.40E-01	1.58E-01	1.76E-01
4.00E-01	1.15E-01	1.21E-01	1.27E-01
5.00E-01	1.01E-01	1.03E-01	1.05E-01
6.00E-01	9.17E-02	9.14E-02	9.12E-02
8.00E-01	7.90E-02	7.72E-02	7.54E-02
1.00E+00	7.04E-02	6.82E-02	6.59E-02

1.02E+00	6.96E-02	6.73E-02	6.51E-02
1.25E+00	6.26E-02	6.03E-02	5.80E-02
1.50E+00	5.69E-02	5.48E-02	5.26E-02
2.00E+00	4.91E-02	4.75E-02	4.59E-02
2.04E+00	4.85E-02	4.70E-02	4.54E-02
3.00E+00	4.00E-02	3.96E-02	3.92E-02
4.00E+00	3.50E-02	3.55E-02	3.60E-02
5.00E+00	3.17E-02	3.30E-02	3.43E-02

Table 3. Mass attenuation coefficients (μ_m) of Eu_2O_3/HDPE composites at filler contents of 20, 40, and 60 wt.% and photon energies of 0.001–5 MeV.

Photon energy (MeV)	Content (wt.%)		
	20	40	60
1.00E-03	2.02E+03	2.15E+03	2.28E+03
1.06E-03	1.72E+03	1.85E+03	1.98E+03
1.13E-03	1.47E+03	1.59E+03	1.72E+03
1.13E-03	1.57E+03	1.79E+03	2.02E+03
1.15E-03	1.73E+03	2.17E+03	2.61E+03
1.16E-03	2.01E+03	2.77E+03	3.53E+03
1.16E-03	2.20E+03	3.15E+03	4.11E+03
1.31E-03	1.75E+03	2.62E+03	3.49E+03
1.48E-03	1.41E+03	2.20E+03	2.99E+03
1.48E-03	1.55E+03	2.47E+03	3.39E+03
1.50E-03	1.49E+03	2.39E+03	3.28E+03
1.61E-03	1.25E+03	2.01E+03	2.77E+03
1.61E-03	1.30E+03	2.11E+03	2.92E+03
1.70E-03	1.14E+03	1.86E+03	2.58E+03
1.80E-03	9.96E+02	1.64E+03	2.28E+03
1.80E-03	1.03E+03	1.70E+03	2.37E+03
2.00E-03	7.93E+02	1.33E+03	1.86E+03
3.00E-03	2.85E+02	4.92E+02	7.00E+02
4.00E-03	1.35E+02	2.38E+02	3.42E+02
5.00E-03	7.56E+01	1.35E+02	1.94E+02
6.00E-03	4.69E+01	8.43E+01	1.22E+02
6.98E-03	3.16E+01	5.71E+01	8.27E+01
6.98E-03	7.65E+01	1.47E+02	2.18E+02
7.29E-03	6.84E+01	1.32E+02	1.95E+02
7.62E-03	6.11E+01	1.18E+02	1.74E+02
7.62E-03	8.16E+01	1.59E+02	2.36E+02
8.00E-03	7.24E+01	1.41E+02	2.09E+02
8.05E-03	7.12E+01	1.38E+02	2.06E+02
8.05E-03	8.16E+01	1.59E+02	2.37E+02
1.00E-02	4.73E+01	9.24E+01	1.38E+02
1.50E-02	1.63E+01	3.19E+01	4.75E+01
2.00E-02	7.70E+00	1.50E+01	2.22E+01
3.00E-02	2.72E+00	5.16E+00	7.61E+00
4.00E-02	1.35E+00	2.47E+00	3.59E+00
4.85E-02	8.74E-01	1.54E+00	2.20E+00
4.85E-02	3.63E+00	7.05E+00	1.05E+01
5.00E-02	3.37E+00	6.53E+00	9.69E+00
6.00E-02	2.16E+00	4.12E+00	6.08E+00
8.00E-02	1.09E+00	2.00E+00	2.91E+00
1.00E-01	6.67E-01	1.16E+00	1.66E+00
1.50E-01	3.12E-01	4.71E-01	6.30E-01
2.00E-01	2.09E-01	2.78E-01	3.47E-01
3.00E-01	1.41E-01	1.61E-01	1.81E-01
4.00E-01	1.16E-01	1.23E-01	1.30E-01
5.00E-01	1.02E-01	1.04E-01	1.06E-01

6.00E-01	9.20E-02	9.21E-02	9.21E-02
8.00E-01	7.92E-02	7.76E-02	7.60E-02
1.00E+00	7.05E-02	6.84E-02	6.63E-02
1.02E+00	6.97E-02	6.76E-02	6.55E-02
1.25E+00	6.27E-02	6.05E-02	5.83E-02
1.50E+00	5.70E-02	5.50E-02	5.29E-02
2.00E+00	4.91E-02	4.76E-02	4.61E-02
2.04E+00	4.86E-02	4.71E-02	4.57E-02
3.00E+00	4.01E-02	3.98E-02	3.94E-02
4.00E+00	3.50E-02	3.56E-02	3.62E-02
5.00E+00	3.18E-02	3.32E-02	3.46E-02

Table 4. Mass attenuation coefficients (μ_m) of Gd_2O_3/HDPE composites at filler contents of 20, 40, and 60 wt.% and photon energies of 0.001–5 MeV.

Photon energy (MeV)	Content (wt.%)		
	20	40	60
1.00E-03	2.03E+03	2.18E+03	2.32E+03
1.06E-03	1.63E+03	1.77E+03	1.91E+03
1.13E-03	1.31E+03	1.44E+03	1.57E+03
1.13E-03	1.34E+03	1.50E+03	1.66E+03
1.15E-03	1.45E+03	1.77E+03	2.09E+03
1.16E-03	1.64E+03	2.18E+03	2.73E+03
1.16E-03	1.78E+03	2.47E+03	3.16E+03
1.31E-03	1.40E+03	2.19E+03	2.99E+03
1.48E-03	1.30E+03	2.04E+03	2.78E+03
1.48E-03	1.42E+03	2.29E+03	3.16E+03
1.50E-03	1.27E+03	2.06E+03	2.85E+03
1.61E-03	1.14E+03	1.85E+03	2.56E+03
1.61E-03	1.19E+03	1.94E+03	2.70E+03
1.70E-03	1.04E+03	1.71E+03	2.39E+03
1.80E-03	9.11E+02	1.51E+03	2.11E+03
1.80E-03	9.39E+02	1.57E+03	2.20E+03
2.00E-03	8.09E+02	1.36E+03	1.91E+03
3.00E-03	2.92E+02	5.06E+02	7.21E+02
4.00E-03	1.39E+02	2.46E+02	3.53E+02
5.00E-03	7.78E+01	1.39E+02	2.01E+02
6.00E-03	4.83E+01	8.71E+01	1.26E+02
6.98E-03	2.95E+01	5.36E+01	7.77E+01
6.98E-03	7.14E+01	1.37E+02	2.04E+02
7.29E-03	6.35E+01	1.22E+02	1.81E+02
7.62E-03	5.65E+01	1.09E+02	1.61E+02
7.62E-03	7.55E+01	1.47E+02	2.18E+02
8.00E-03	7.41E+01	1.44E+02	2.14E+02
8.05E-03	6.61E+01	1.29E+02	1.91E+02
8.05E-03	7.57E+01	1.48E+02	2.20E+02
1.00E-02	4.86E+01	9.50E+01	1.42E+02
1.50E-02	1.68E+01	3.29E+01	4.90E+01
2.00E-02	7.94E+00	1.54E+01	2.29E+01
3.00E-02	2.80E+00	5.33E+00	7.86E+00
4.00E-02	1.39E+00	2.55E+00	3.71E+00
4.85E-02	8.42E-01	1.48E+00	2.11E+00
4.85E-02	8.34E-01	1.46E+00	2.09E+00
5.00E-02	3.41E+00	6.60E+00	9.80E+00
6.00E-02	2.20E+00	4.21E+00	6.21E+00
8.00E-02	1.12E+00	2.05E+00	2.99E+00
1.00E-01	6.81E-01	1.19E+00	1.70E+00
1.50E-01	3.17E-01	4.81E-01	6.45E-01
2.00E-01	2.12E-01	2.83E-01	3.54E-01

3.00E-01	1.42E-01	1.62E-01	1.83E-01
4.00E-01	1.16E-01	1.23E-01	1.30E-01
5.00E-01	1.02E-01	1.04E-01	1.06E-01
6.00E-01	9.20E-02	9.20E-02	9.20E-02
8.00E-01	7.91E-02	7.74E-02	7.57E-02
1.00E+00	7.04E-02	6.82E-02	6.60E-02
1.02E+00	6.96E-02	6.74E-02	6.51E-02
1.25E+00	6.26E-02	6.03E-02	5.79E-02
1.50E+00	5.69E-02	5.47E-02	5.25E-02
2.00E+00	4.90E-02	4.74E-02	4.58E-02
2.04E+00	4.85E-02	4.69E-02	4.54E-02
3.00E+00	4.00E-02	3.96E-02	3.92E-02
4.00E+00	3.50E-02	3.55E-02	3.60E-02
5.00E+00	3.18E-02	3.31E-02	3.44E-02

Table 5. Comparative μ_m, μ, and HVL values of Sm_2O_3/HDPE, Eu_2O_3/HDPE, and Gd_2O_3/HDPE composites with other common Pb-free HDPE composites (Bi_2O_3/HDPE, WO_3/HDPE, and Fe_2O_3/HDPE) at filler contents of 20, 40, and 60 wt.% and photons energies of 0.1, 0.5, 1, and 5 MeV.

Parameter	Photon energy (MeV)	Content (wt.%)	Sm_2O_3	Eu_2O_3	Gd_2O_3	WO_3	Bi_2O_3	Fe_2O_3
μ_m (cm^2/g)	0.1	20	0.64220	0.68120	0.66690	0.84760	1.17000	0.19890
		40	1.11300	1.19000	1.16200	1.52300	2.16800	0.22580
		60	1.58300	1.70000	1.65700	2.19900	3.16600	0.25270
	0.5	20	0.10120	0.10170	0.10160	0.10500	0.11110	0.09659
		40	0.10290	0.10380	0.10380	0.11060	0.12270	0.09372
		60	0.10450	0.10600	0.10590	0.11620	0.13430	0.09084
	1.0	20	0.07039	0.07041	0.07052	0.07123	0.07235	0.07032
		40	0.06815	0.06819	0.06842	0.06984	0.07208	0.06801
		60	0.06591	0.06597	0.06632	0.06845	0.07181	0.06570
	5.0	20	0.03173	0.03176	0.03183	0.03202	0.03267	0.03043
		40	0.03302	0.03308	0.03320	0.03358	0.03490	0.03041
		60	0.03431	0.03440	0.03458	0.03515	0.03713	0.03039
μ (cm^{-1})	0.1	20	0.74152	0.78380	0.76732	0.97421	1.35326	0.22595
		40	1.63801	1.73581	1.69478	2.21545	3.20462	0.31896
		60	3.21155	3.38629	3.29992	4.35574	6.48148	0.47185
	0.5	20	0.11685	0.11702	0.11690	0.12068	0.12850	0.10973
		40	0.15144	0.15141	0.15139	0.16089	0.18137	0.13239
		60	0.21201	0.21115	0.21090	0.23017	0.27494	0.16962
	1.0	20	0.08128	0.08102	0.08114	0.08187	0.08368	0.07988
		40	0.10030	0.09947	0.09979	0.10159	0.10654	0.09607
		60	0.13372	0.13141	0.13208	0.13558	0.14701	0.12268
	5.0	20	0.03664	0.03654	0.03662	0.03680	0.03779	0.03457
		40	0.04860	0.04825	0.04842	0.04885	0.05159	0.04296
		60	0.06961	0.06852	0.06887	0.06962	0.07601	0.05674
HVL (cm)	0.1	20	0.93476	0.88434	0.90334	0.71150	0.51220	3.06767
		40	0.42316	0.39932	0.40899	0.31287	0.21630	2.17311
		60	0.21583	0.20469	0.21005	0.15913	0.10694	1.46901
	0.5	20	5.93187	5.92342	5.92949	5.74347	5.39405	6.31700
		40	4.57708	4.57798	4.57846	4.30832	3.82176	5.23569
		60	3.26946	3.28280	3.28662	3.01150	2.52108	4.08652
	1.0	20	8.52827	8.55576	8.54278	8.46644	8.28306	8.67689
		40	6.91096	6.96868	6.94599	6.82274	6.50569	7.21496
		60	5.18371	5.27477	5.24808	5.11229	4.71495	5.65022
	5.0	20	18.91917	18.96761	18.92669	18.83400	18.34341	20.05123
		40	14.26353	14.36499	14.31460	14.19001	13.43639	16.13578
		60	9.95798	10.11560	10.06514	9.95552	9.11878	12.21518
